# Surgery in times of COVID-19—recommendations for hospital and patient management

**DOI:** 10.1007/s00423-020-01888-x

**Published:** 2020-05-08

**Authors:** S. Flemming, M. Hankir, R.-I. Ernestus, F. Seyfried, C.-T. Germer, P. Meybohm, T. Wurmb, U. Vogel, A. Wiegering

**Affiliations:** 1 Department of General, Visceral, Transplantation, Vascular and Pediatric Surgery, University Hospital Wuerzburg, Oberduerrbacher Str. 6, 97080 Wuerzburg, Germany; 2 Department of Neurosurgery, University of Wuerzburg, Wuerzburg, Germany; 3 Department of Anaesthesia and Critical Care, University of Wuerzburg, Wuerzburg, Germany; 4 Institute for Clinical Microbiology and Infectiology, Julius-Maximilians-University Wuerzburg, Wuerzburg, Germany; 5 Hospital Infection Control Team at the University Hospital Wuerzburg, Wuerzburg, Germany; 6 Department of Biochemistry and Molecular Biology, Theodor Boveri Institute, University of Wuerzburg, Wuerzburg, Germany

**Keywords:** SARS-CoV-2, COVID-19, Surgery

## Abstract

**Background:**

The novel coronavirus disease 2019 (COVID-19), caused by severe acute respiratory syndrome coronavirus 2 (SARS-CoV-2), has escalated rapidly to a global pandemic stretching healthcare systems worldwide to their limits. Surgeons have had to immediately react to this unprecedented clinical challenge by systematically repurposing surgical wards.

**Purpose:**

To provide a detailed set of guidelines developed in a surgical ward at University Hospital Wuerzburg to safely accommodate the exponentially rising cases of SARS-CoV-2 infected patients without compromising the care of emergency surgery and oncological patients or jeopardizing the well-being of hospital staff.

**Conclusions:**

The dynamic prioritization of SARS-CoV-2 infected and surgical patient groups is key to preserving life while maintaining high surgical standards. Strictly segregating patient groups in emergency rooms, non-intensive care wards and operating areas prevents viral spread while adequately training and carefully selecting hospital staff allow them to confidently and successfully undertake their respective clinical duties.

## Introduction

At the end of 2019, several mysterious pneumonia cases of suspected viral origin were reported for the first time in Wuhan, China. Detailed virological and genomic analyses of patient swab samples subsequently traced these cases to a novel type of corona virus termed SARS-CoV-2, which is responsible for the clinical condition and now global pandemic referred to as “COVID-19” [1].

Coronaviruses are single-stranded RNA viruses that are classified into 4 types [2]. SARS-CoV-2, as well as the previously characterized SARS-CoV which caused the 2002/2003 SARS pandemic, belongs to the genus of beta coronaviruses [1, 2]. These viruses show 82% homology in their genomic sequences [3] and attach to host cells through their spike proteins (SARS-2-S and SARS-S, respectively) binding to angiotensin-converting enzyme 2 (ACE2) as a receptor [4]. Viral fusion with the host cell and then infection follows as a result of cellular cysteine and serine protease-mediated cleavage of SARS-2-S and SARS [4]. Importantly, ACE2 is found not only in cells of the cardiopulmonary system (lungs, heart, endothelium, kidney), but also in epithelial cells lining the gastrointestinal tract [5, 6]. This expression pattern of ACE2 has implications for the clinical symptoms of patients suffering from SARS-CoV-2 infection. During the SARS outbreak in 2002/2003, patients not only showed pulmonary symptoms, but also presented with gastrointestinal complaints (16–73% of cases) [2, 5]. Covid-19 patients also appear to have gastrointestinal issues in up to 10% of cases [7–10].

Due to the rapid global spread of SARS-CoV-2 [1], healthcare systems and their workers worldwide face tremendous challenges, and surgery as a discipline is by no means an exception. Preparations for the developing crisis have initially centred on the provision of intensive care capacities to ventilate seriously ill patients and the maintenance of an adequate supply of protective equipment for medical personnel. As a result, politicians have demanded postponement or even outright cancellation of all elective operations [11, 12]. So far, these measures have failed to take into account SARS-CoV-2 positive patients whose main symptoms of disease are not specific to COVID-19 (e.g. respiratory symptoms, fever) and are thus diagnosed as asymptomatic or oligosymptomatic but with surgical disease (e.g. acute appendicitis, acute limb ischemia, distal radius fracture). These patients must therefore be treated primarily surgically without the need for COVID-19 designation or allocation (e.g. in intensive care medicine).

Surgery is a basic pillar of medical care, which results in the following challenges for everyday clinical practice:

- Prioritization of surgical interventions
- Establishment of SARS-CoV-2 and non-SARS-CoV-2 emergency rooms
- Establishment of a SARS-CoV-2 surgical non-intensive care ward
- Establishment of a surgical SARS-CoV-2 operating area
- Necessary precautions when using certain surgical techniques

This article is based on real-time experiences from a Surgical Department at the University Hospital Würzburg, Germany, in planning and preparing clinical pathways to ensure high-quality surgical care during the COVID-19 pandemic. These pathways were developed and established in consultation with colleagues from the departments of anaesthesiology, intensive care medicine, microbiology and virology and the specialist team for hospital hygiene.

Since the COVID-19 outbreak is of a very dynamic nature, the following recommendations need to be reevaluated and adapted according to the current situation. This needs to be assessed at least on a daily basis by an interdisciplinary team consisting of members of anaesthesiology, intensive care, internal medicine, surgery, infectiology and hospital hygiene. It may be necessary that due to the high dynamics of events, multi-interdisciplinary exchange needs to take place to modify or adapt existing concepts on demand [13].

## Prioritization of operational interventions

In general, the federal and state governments have mandated that non-urgent surgical interventions should be postponed or suspended [11, 12, 14]. On the other hand, emergency surgical care and the operative therapy of tumour patients and patients with urgent indications should continue. It should be pointed out, however, that it is still entirely unclear when routine operations can resume and whether patients who do not require immediate surgical attention should be granted increased priority if the need arises. So far, the German Society of Epidemiology estimates that the pandemic will last for at least 3 months depending on the success of current restrictions in social life [15].

In order to meet these requirements, it is recommended to create a prioritization list of the operations performed in the respective surgical department. This prioritization should take place from both a medical and logistical point of view and therefore requires close coordination between the operational partners in order to continue to ensure adequate perioperative treatment quality. In addition to the intensive care unit capacity for non-COVID-19 patients, personnel and material resources should also be taken into account in the context of pandemic planning [14, 16, 17].

The decision whether a surgical therapy can be postponed and, if so, in which time frame, must be made by colleagues who have a high level of expertise in this specific area. It is the duty of care of each operative department to define this for their respective patient group. It should consider whether postponement of an operation leads to a significantly worse outcome, an exacerbation of the underlying disease or an unacceptable reduction in the quality of life of the patient. The prioritization list also includes the period in which the patient should present themselves for evaluation and preparation for surgery or if telemedical care is possible. Thus, the list can also serve as the basis for outpatient management in order to achieve efficient and aligned patient prioritization.

At the University Hospital Würzburg, a list of priorities in four urgency levels was drawn up against the background of the abovementioned aspects (see Table 1) based on recommendations of international surgical societies [14, 18–20]. Urgency level I includes diseases that require immediate surgical intervention (within 2 weeks; e.g. colorectal cancer with local complications, symptomatic carotid stenosis, inguinal hernia with signs of incarceration). Urgency level II includes high-priority operations that should be done within 2–4 weeks. These are not just limited to tumour operations, but also include benign clinical conditions with a high level of suffering or expected (serious) consequences if the operation is not performed (e.g. colorectal liver metastases, symptomatic chest or stomach, hernias with recurrent incarceration symptoms, conservative therapy refractory symptomatic anal fissure). Urgency level III includes all operations that allow a delay of 4–8 weeks (e.g. chronic recurrent sigmoid diverticulitis). Urgency level IV includes all other elective procedures (e.g. plastic-aesthetic procedures, ostomy relocation without local complications, functional rectal diseases).

**Table 1:** Representative examples of surgical interventions (colorectal surgery) according to their urgency (level I–IV). Prioritization should be subject- and intervention-specific

Priority level	Disease (examples)	Recommended time of operation (weeks)	Priority of outpatient presentation
I	Trauma, bleeding (cancer, inflammation, haemorrhoids, etc.), after-bleeding, septic focus/abscess, perforation, toxic megacolon (ulcerative colitis, *Clostridium difficile* infection) Colorectal cancer with local complications (e.g. bleeding and stenosis) Complicated antibiotic-refractory diverticulitis Crohn’s ileitis with local complications (e.g. entero-cutaneous fistula, retroperitoneal fistula, abscess) Acute appendicitis	0–2	Immediately
II	Colorectal cancer without neo-adjuvant treatment Rectal cancer with neo-adjuvant treatment (if applicable prolonged interval between neo-adjuvant treatment and operation) Therapy-refractory ulcerative colitis Anal carcinoma Therapy-refractory anal fissure	2–4	Next working day
III	Chronic and recurrent diverticulitis Crohn’s ileitis without local complications Rectal adenoma (trans-anal excision, trans-anal microsurgery)	4–12	1–2 weeks
IV	Symptomatic haemorrhoids (except bleeding ➔ priority level I) Ileostomy/colostomy reversal without local complications (with local complications ➔ priority level II) Rectal prolapse, obstructed defecation syndrome, pilonidal disease	> 12	No physical appointment, telemedical care

The prioritization concepts of operations and the associated outpatient appointment for planning the operation or clinical patient evaluation should be communicated to the outpatient referring colleagues immediately, so that appropriate patient care is passed on between the outpatient and in-hospital sector.

## Establishment of SARS-CoV-2 and non-SARS-CoV-2 emergency rooms

To avoid the transmission of SARS-CoV-2 between patients or from patients to healthcare workers and physicians, and to conserve personal protective equipment (PPE), emergency rooms (ER) should be divided into infectious (SARS-CoV-2 suspected and confirmed patients; COVID area) and non-infectious parts (non-COVID area). Checkpoints at the entrance of the ER are set up to assess patients for symptoms, provide them with surgical masks before entering the hospital and guide them to COVID or non-COVID areas. The assessment of patients regarding SARS-CoV-2/COVID-19 includes a short questionnaire about typical symptoms for COVID-19, potential contact to SARS-CoV-2 positive tested persons, residence in a nursing/senior-citizen home with evidenced outbreak of COVID-19 and wireless body temperature monitoring. Medical students with prior specialist training can perform this initial assessment.

This separation of positive or potentially positive SARS-CoV-2 patients and non SARS-CoV-2 patients has to be performed also for patients with an asymptomatic or oligosymptomatic course of disease entering primarily the ER because of surgical diseases and problems, respectively.

Furthermore, the COVID ER area should have an operation/intervention section to perform surgical trauma/wound management as well as reduction of traumatic and fracture dislocations.

Every patient in the COVID area must be swabbed for SARS-CoV-2 PCR testing. Depending on the test result and further medical/surgical treatment, the patient will then be transferred to an operation room, intensive care unit or hospital ward, which are also separated into SARS-CoV2 or non-SARS-CoV-2 areas. If it is medically indicated, patients can be also discharged from the hospital in accordance with local restrictions.

## Establishment of a SARS-CoV-2 surgical non-intensive care ward

As mentioned above, there will be both COVID-19 sick and SARS-CoV-2 positive patients, who, however, only show an oligo- or even asymptomatic course of disease. If these patients immediately need emergency or urgent surgery, no or only short-term intensive medical treatment may be necessary and postoperative surgical follow-up care in the non-intensive care ward is indicated. Based on this, it is recommended to establish surgical SARS-CoV-2 wards in consultation with the department of infectious diseases and hospital hygiene [21]. On March 20, 2020, an interdisciplinary surgical SARS-CoV-2 ward was established at the Center for Operative Medicine of the University Hospital Würzburg, in which non-intensive care patients with suspected or evidence of SARS-CoV-2 infection in the departments of gastrointestinal surgery, vascular surgery, trauma surgery, cardiac/thoracic surgery and urology are placed under perioperative surveillance and care.

**Fig. 1:**
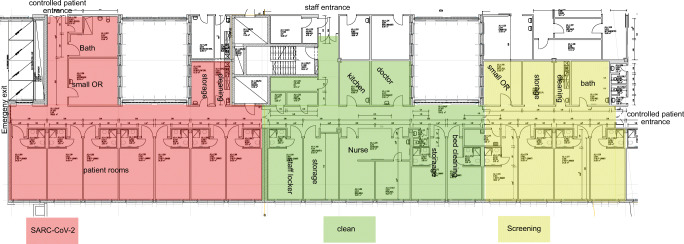
Structure and structuring of the surgical SARS-CoV-2 bedside unit (modified from construction drawing of “Zentrum für Operative Medizin, University Hospital Wuerzburg”), which is divided into 3 areas: infectious area (red), for SARS-CoV-2 positive patients, potentially infectious area (yellow) for suspected patients with SARS-CoV-2 and the clean area (green). Patient access is controlled via the semi-infectious area. There is separate access to the station for medical staff to enter the clean station area directly

In cooperation with the hospital infection control team and facility management, a non-intensive care ward was repurposed and newly set up. This included the establishment of 3 discrete areas: SARS-COV-2 positive area (infectious area), SARS-CoV-2 suspicious area (potentially infectious area) and non-infectious area (nursing support point, doctor’s room, staff changing room including sanitary facilities, common room) (Fig. 1).

Suspicious cases are primarily recorded in the designated area. Each patient is carefully isolated here to avoid potential transmission and if the test is negative, the patient is then transferred to a non-infectious hospital ward. Conversely, if the result is positive, the patient is moved to the SARS-CoV-2 positive area. It is critical here that cohort isolation under certain conditions is made possible in the SARS-CoV-2 positive area, which allows central entry and exit. In addition to the structural requirements, an interdisciplinary nursing and medical team was put together, whose role is to exclusively take care of these patients. Due to the interdisciplinary patient population, the isolation measures and the associated physical and psychological stress for the employees, individuals were selected according to the following characteristics: proven technical expertise, intensive medical experience, calm and thoughtful work ethic and high level of teamwork and adaptability. Before the official announcement, each potential team member was personally asked whether he/she felt up to the task and would be willing to take on the responsibility. As a result, a motivated team focused on the challenge was created right from the onset. This team was jointly prepared for the challenges and was given intensive hygiene training (handling SARS-CoV-2 and the necessary personal protective equipment including correct donning and doffing).

The establishment of this special ward significantly reduces the risk of transmission between patients and also minimizes the risk for medical personnel. In addition, unnecessary intensive care unit capacities can be avoided due to the need for isolation rather than due to medical indications.

## Establishment of a SARS-CoV-2 operation area

In addition to a surgical SARS-CoV-2 non-intensive care unit, a special operating area for SARS-CoV-2 positive patients or suspected cases should also be established based on the recommendation of international surgical societies [18, 20, 22]. The aim here is to also minimize the spread and thus the risk of transmission to patients and medical staff. The development of this surgical area should again involve members of the infection control team, anaesthesiology and surgical technical assistant (OTAs)/nursing teams. According to the recommendations of Brindle et al. and the American College of Surgeons, the following should be considered [16, 18]:

1. Ideally, the operating area should have anterooms with a negative pressure system (CAVE: that do not use an overpressure system) and separate access, and be located away from high-traffic areas. The anteroom should be used for donning and doffing of personal protective equipment and as a storage place for medication and surgical materials that may be needed during the operation. In the absence of an anteroom, an extra cordoned off/marked area should be reserved for these activities.
2. Materials or objects not essential for the operation (e.g. pens, telephones, keys) should remain outside the SARS-CoV-2 operating area. The materials required for the operation should all be disinfected and immediately disposed of after use in accordance with the hygiene guidelines (guidelines of local and governmental authorities, in-house regulations for hospital hygiene).
3. Entering and leaving the operating room should be kept to an absolute minimum. For this reason, an additional “jumper” is necessary in the anteroom to pass any missing materials into the operating room.
4. The patient should not be moved to a post-anaesthesia recovery room. The patient should be followed up in the operating room and then transferred directly to the SARS-CoV-2 surgical ward, intermediate care station or intensive care unit.
5. The patient’s route between the operating room and the ward should be covered as quickly and directly as possible and has priority over other transport. If the patient is not intubated, a surgical mask for the patient is recommended.
6. In order to achieve the shortest possible exposure time for medical personnel, appropriate surgical techniques should be used [23].
7. Intubation, ventilation and extubating should take place within the framework of currently valid recommendations of anaesthesiological associations [13, 17].
8. A meticulous list of the people involved in the surgical intervention should be made, starting with entry, in order to be able to guarantee disease monitoring.

## Surgical techniques to reduce transmission and exposure risk

The pandemic transmission of SARS-CoV-2 happens by aerosolized viral particles; however, it remains unclear if faecal-oral transfer is also possible [5] and if the virus is able to spread in the peritoneal cavity or other bodily fluids. Negative pressure systems and laminar air flow should significantly reduce the number of virus particles in the operation room [24, 25].

A large number of surgical interventions are performed by minimally invasive approaches using carbon dioxide insufflation (CO_2_). Especially in the field of gastrointestinal surgery, minimally invasive surgery is the gold standard of many diseases and, thus, it is crucial to establish and to maintain a pneumoperitoneum increasing the potential risk of exposure to aerosolized viral particles. Therefore, an uncontrolled release of the pneumoperitoneum should be prevented, and filter systems or closed circuits (e.g. used for pressurized intraperitoneal aerosol chemotherapy (PIPAC)) should be used [12, 26]. Furthermore, CO_2_ pressure and flow should be kept to a minimum. However, since previous studies have shown that viral and bacterial aerosols can be detected in both laparoscopic and open surgical operations [27], a surgical aspirator/smoke evacuation device should be also used in open procedures. Energy devices and electrical instruments should be utilized on the lowest energy level to avoid unnecessary production of smoke and aerosols.

So far, there is no broad evidence for these recommendations and further studies are urgently needed; however, the safest approach to avoid SARS-CoV-2 transmission may be the one that reduces operation time and is the most familiar to the operation team.
